# Unraveling the roles of aromatic cluster side-chain interactions on the structural stability and functional significance of psychrophilic *Sphingomonas* sp. glutaredoxin 3

**DOI:** 10.1371/journal.pone.0290686

**Published:** 2023-08-31

**Authors:** Trang Van Tran, Trang Hoang, Sei-Heon Jang, ChangWoo Lee

**Affiliations:** Department of Biomedical Science and Center for Bio-Nanomaterials, Daegu University, Gyeongsan, South Korea; Weizmann Institute of Science, ISRAEL

## Abstract

This study investigates the impact of aromatic cluster side-chain interactions in Grx3 (SpGrx3) from the psychrophilic Arctic bacterium *Sphingomonas* sp. Grx3 is a class I oxidoreductase with a unique parallel arrangement of aromatic residues in its aromatic cluster, unlike the tetrahedral geometry observed in Trxs. Hydrophilic-to-hydrophobic substitutions were made in the aromatic cluster, in β1 (E5V and Y7F), adjacent β2 (Y32F and Y32L), both β1 and β2 (E5V/Y32L), and short α2 (R47F). The hydrophobic substitutions, particularly those at or near Tyr7 (E5V, Y7F, Y32F, and R47F), increased melting temperatures and conformational stability, whereas disrupting β1-β2 interactions (Y32L and E5V/Y32L) led to structural instability of SpGrx3. However, excessive hydrophobic interactions (Y7F and E5V/Y32L) caused protein aggregation at elevated temperatures. All mutations resulted in a reduction in α-helical content and an increase in β-strand content. The R47F mutant, which formed dimers and exhibited the highest β-strand content, showed increased conformational flexibility and a significant decrease in catalytic rate due to the disturbance of β1-α2 interactions. In summary, the configuration of the aromatic cluster, especially Tyr7 in the buried β1 and Arg47 in the short α2, played crucial roles in maintaining the active conformation of SpGrx3 and preventing its protein aggregation. These modifications, reducing hydrophobicity in the central β-sheet, distinguish Grx3 from other Trx-fold proteins, highlighting evolutionary divergence within the Trx-fold superfamily and its functional versatility.

## Introduction

Grxs are small redox proteins with approximately 85–150 amino acids that use GSH as an electron donor [1, 2]. Grxs play a crucial role in various cellular processes, including antioxidant defense and cellular redox homeostasis [2, 3]. Grxs share a typical Trx-fold consisting of a four-stranded β-sheet sandwiched by three to four α-helices, with the β-strand topology of 2134 [4, 5]. The Trx-fold features an N-terminal βαβ motif and a C-terminal ββα motif, connected by a short α-helix and cis-proline loop [6, 7]. Grxs are divided into at least four classes with classes I and II the most widespread [1]. Class I Grxs have a monothiol or dithiol [CXXC/S] active site and are glutathione-dependent oxidoreductases [8]. Class II Grxs, which have a monothiol [CGFS] active site, are redox-inactive and serve as Fe-S cluster transferases [8–10].

**Fig 1:**
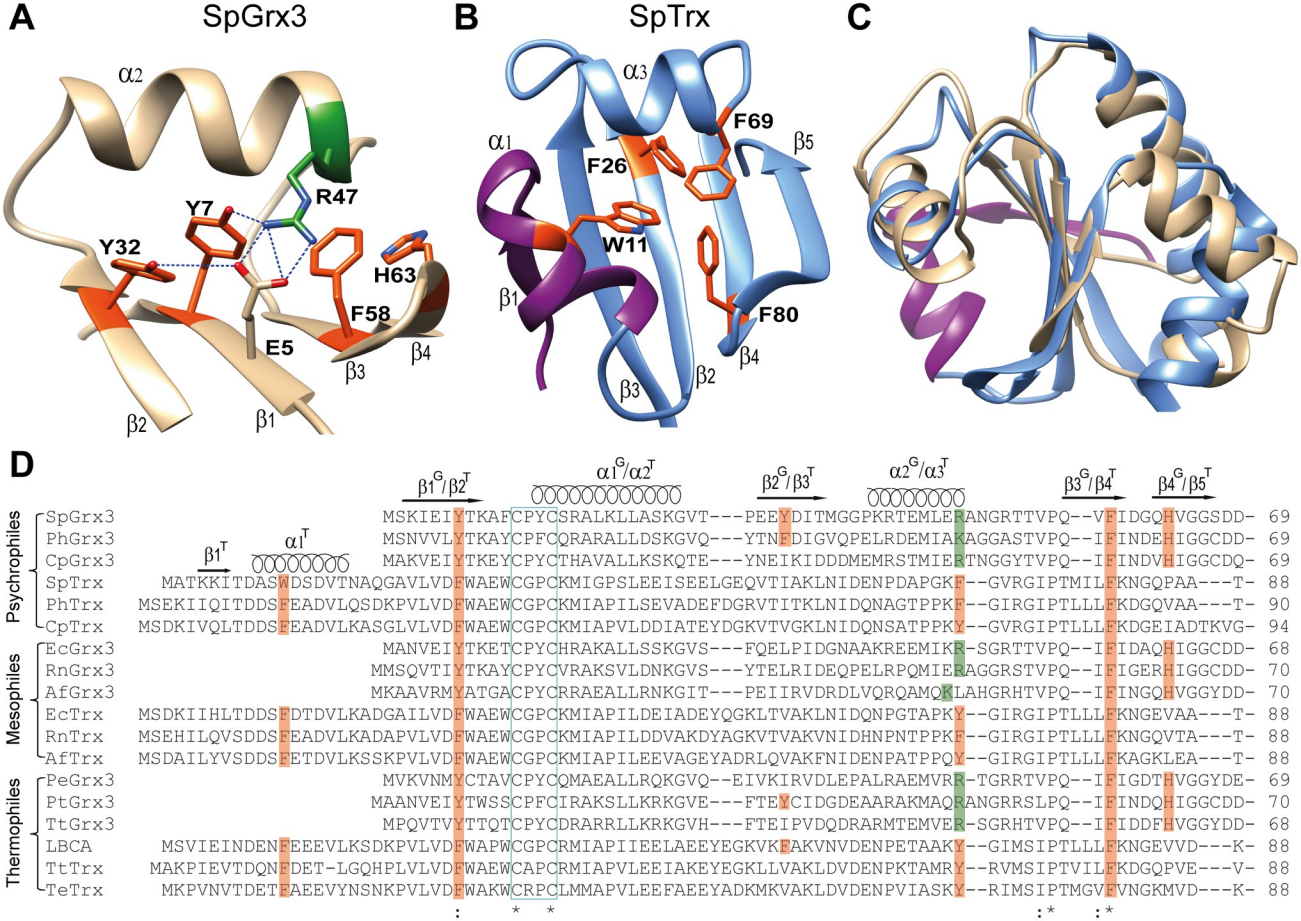
Structure and sequence comparison. (A) Enlarged view of the aromatic cluster side of SpGrx3 and (B) SpTrx. (C) Overlap structures of SpGrx3 (gold) and SpTrx (blue), highlighting the additional α-helix and β-strand in SpTrx (purple). (D) Sequence comparison of Trx and Grx3 members with different habitat temperatures. The active site motif is denoted by the cyan box. The α-helices and β-strands of Grx3 and Trx are labeled with superscripts G and T, respectively. The sequences examined include SpGrx3 (WP_010217562.1), PhGrx3 (YP_338909.1), CpGrx3 (WP_011045119.1), SpTrx (WP_010164143.1), PhTrx (WP_008109071), CpTrx (KGJ92637.1), EcGrx3 (1FOV), RnGrx3 (WP_008217797.1), AfGrx3 (WP_035195182.1), EcTrx (2TRX), RnTrx (WP_008222599.1), AfTrx (AQS23897.1), PeGrx3 (TXF13737.1), PtGrx3 (WP_191130001.1), TtGrx3 (QGU33019.1), LBCA (4BA7), TtTrx (BAW01746.1), and TeTrx (EEU61346.1).

The Trx-fold, a highly conserved structural motif found in the Trx-fold superfamily [6, 11], exhibits a distinctive arrangement of its hydrophobic core. This core is divided into two clusters on the central β-sheet: one composed of aliphatic residues (aliphatic cluster) and the other composed of aromatic residues (aromatic cluster), which is flanked by the short α-helix [12, 13]. Grx3, a class I oxidoreductase, exhibits a unique aromatic cluster configuration with a reduction of one α-helix and one β-strand compared to Trx (Fig 1A–1C). While the aromatic cluster of Trx had a tetrahedral geometry of aromatic residues with nearly identical side-chain conformations [14, 15], the aromatic cluster of Grx3 exhibits a parallel arrangement of two conserved aromatic residues in the central β-sheet (β1 and β3) and a dominant His residue in β4 (Fig 1A). Although His is typically classified as a basic amino acid, it has an aromatic motif with a planar ring structure in its imidazole side chain [16]. Additionally, Grx3 diverges from Trx in its aromatic cluster configuration through substitutions within the β1-strand and short α2-helix. In Grx3, Tyr and Arg/Lys residues replace Phe residues in the β1-strand and short α2-helix, respectively (Fig 1D). However, the effect of these distinct aromatic cluster configurations on the stability and function of Grx3 has not been fully elucidated.

This study investigates the roles of mutated side-chain interactions in the aromatic cluster in Grx3 (SpGrx3) from the psychrophilic Arctic bacterium *Sphingomonas* sp. PAMC 26621 [17], in comparison to Trx (SpTrx) from the same bacterium [12]. SpGrx3 displays a parallel configuration of aromatic residues (Tyr7, Tyr32, Phe58, and His63), with each β-strand containing one aromatic residue, in contrast to the tetrahedral arrangement of aromatic residues (Trp11, Phe26, Phe69, and Phe80) observed in SpTrx (Fig 1A and 1B) [12]. Interestingly, Tyr32 in β2 distinguishes SpGrx3 from other Grx3 members, which typically have branched aliphatic residues such as Val, Leu, or Ile (Fig 1D). The structural model of SpGrx3 shows aromatic–aromatic interactions and hydrogen bonds between the β1-β2 strands, and salt bridges and hydrogen bonds between the β1-α2 strand-helix (Fig 1A). The Protein Interactions Calculator (PIC) predicted aromatic–aromatic interactions between Tyr7 in β1 and Tyr32 in β2, with a distance of 4.8 Å and an angle of 66.1° (Fig 1A). We used hydrophilic-to-hydrophobic site-directed mutagenesis to introduce mutations in β1 (E5V and Y7F), adjacent β2 (Y32F and Y32L), as well as in both β1 and β2 (E5V/Y32L), and the short α2 (R47F). This study sheds light on the crucial roles of the aromatic cluster configuration with reduced hydrophobicity in influencing the stability and activity of SpGrx3.

## Materials and methods

### Gene cloning and site-directed mutagenesis

The strain *Sphingomonas* sp. PAMC 26621, isolated from the Svalbard Island in the Arctic Ocean, was kindly provided by the Polar and Alpine Microbial Collection at the Korea Polar Research Institute (Incheon, South Korea) [18]. The polymerase chain reaction (PCR) was employed to amplify the 258-bp *grxC* (*spgrx3*) gene (NCBI ID: NZ_AIDW01000021.1:c56439-56182) from the genome of *Sphingomonas* sp. PAMC 26621, which was subsequently subcloned into a TA vector (Enzynomics, Daejeon Korea). After digestion with *Nde* I and *EcoR* I enzymes TA–*spgrx3* construct, the pET28–*spgrx3* construct was inserted into a pET28 vector (Novagen, Madison, WI, USA) and then introduced into *Escherichia coli* BL21 (DE3) through transformation. Site-directed mutagenesis of the pET28–*spgrx3* construct was performed using *pfu* polymerase and PCR primers listed in S1 Table. After PCR amplification, the products were subjected to *Dpn* I digestion at 37°C for 1 h to remove the template plasmids. The resulting PCR products were then transformed into *E*. *coli* BL21 (DE3). The DNA sequences of both wild-type (WT) and mutant plasmids were validated through DNA sequencing analysis.

### Bioinformatics

The structural models were generated using AlphaFold [19]. The confidence of the predicted structure was evaluated using the pLDDT (predicted local-distance difference test) metric, with regions exhibiting pLDDT > 90 indicating high accuracy in the predicted structure [19]. For SpGrx3, the pLDDT score was 92.9, TtGrx3 had a pLDDT score of 94.1, and SpTrx had a pLDDT score of 95.7. ChimeraX software (version 1.6) was used for protein structure visualization and analysis of amino acid interactions [20]. The sequences were aligned using Clustal Omega [21]. The angle (θ) and distance (D) of the aromatic–aromatic and cation-π interactions were predicted and calculated using a PIC server [22].

### Expression and protein purification

*E*. *coli* BL21 (DE3) was cultured overnight in Luria–Bertani (LB) medium supplemented with 100 μg/mL kanamycin from a single colony. After inoculating the overnight culture into 200 mL of freshly prepared LB medium, the cells were incubated at 37°C until they reached an optical density of 0.6–0.8. Then, the LB medium was supplemented with isopropyl β-D-1-thiogalactopyranoside (1 mM final concentration). The culture was grown for an additional 24 h at 25 °C. Following centrifugation, the cells were collected and subjected to a wash with buffer A, which consisted of 50 mM Tris·HCl, 50 mM NaCl, 5 mM imidazole, and 0.1 mM ethylenediaminetetraacetate (EDTA). The cell pellets containing SpGrx3 were resuspended in buffer A and subjected to sonication on ice. Following centrifugation at 10,000 × *g* for 30 min at 4°C, the resulting supernatant was collected and loaded onto a 1-mL HisTrap column (GE Healthcare, Piscataway, NJ, USA) that had been pre-equilibrated with buffer A. The non-specific proteins were eliminated through column washing using buffer A supplemented with 20 mM imidazole, followed by elution of the target proteins using buffer B containing 300 mM imidazole. All fractions containing SpGrx3 were then loaded onto a 1-mL Capto Q column using an AKTA explorer system (GE Healthcare). The column was pre-equilibrated with buffer C (50 mM Tris·HCl and 50 mM NaCl, pH 8.0). Fractions containing the target proteins were desalted using a HiTrap desalting column and combined in buffer D (100 mM Tris·HCl, pH 8.0). The purified proteins were stored in 5% glycerol and kept at −80°C.

The molecular weights (MW) of the proteins were determined through size exclusion chromatography using a Superdex 200 prep grade XK16 column. The experiment was conducted in buffer C (50 mM Tris·HCl and 50 mM NaCl, pH 8.0). To calculate the molecular weights, a protein standard mix from Sigma was employed, including the following proteins and their respective molecular weights: β-amylase (sweet potato, 200 kDa), alcohol dehydrogenase (yeast, 150 kDa), albumin (bovine serum, 66 kDa), carbonic anhydrase (bovine erythrocytes, 29 kDa), and cytochrome c (horse heart, 12.4 kDa).

### Fluorescence spectroscopy analysis

Fluorescence spectroscopy was performed using a Scinco FS-2 fluorescence spectrometer (Seoul, South Korea). With no Trp residues present in SpGrx3, an excitation wavelength of 275 nm was used to target its three Tyr residues, and the emission range was set to 285–350 nm. The fluorescence intensity of WT was set as 100%.

The effect of acrylamide on protein fluorescence (approximately 240 μg of each protein) was determined by analyzing the quenching of fluorescence with increasing acrylamide concentrations (0–0.25 M) in buffer D after 2 min incubation at 25°C. The obtained results were presented as Stern–Volmer plots, which illustrate the relationship between the ratio of intrinsic fluorescence intensity in the absence of acrylamide (F_0_) and the fluorescence intensity in the presence of acrylamide (F). The analysis of the Stern–Volmer plot demonstrated a positive correlation between F_0_/F and [Q]. This relationship was further utilized in fitting the modified Stern–Volmer equation (F_0_/F = 1 + *K*_*D*_ [Q]), where [Q] represents the acrylamide concentration and *K*_D_ corresponds to the Stern–Volmer constant. The *K*_D_ value was determined by calculating the slope of the linear region of the quenching curve.

The thermal denaturation of SpGrx3 WT and mutant proteins was assessed by measuring the 4,4′-Dianilino-1,1′-binaphthyl-5,5′-disulfonic acid (bis-ANS) fluorescence. Protein samples (13 μg) in buffer D were subjected to temperature incubation at different temperatures (4, 25, 40, 50, and 60°C) for 1 h. Following the incubation, the protein samples were mixed with a reaction mixture consisting of 10 μM bis-ANS in buffer D and incubated for an additional 20 min. Fluorescence spectra of the samples were acquired at a temperature of 25°C using an excitation wavelength of 385 nm and an emission range of 400–600 nm. The highest bis-ANS fluorescence intensity of each protein was set as 100%.

### Urea denaturation curves

The urea-induced equilibrium denaturation curves of SpGrx3 WT and mutants were studied by measuring the fluorescence of tyrosine residues on a Scinco FS-2 fluorescence spectrometer (Seoul, South Korea). SpGrx3 proteins (100 μg) were incubated with different concentrations of urea (0–9 M) in buffer E (100 mM potassium phosphate, and 150 mM NaCl, pH 7.0) at 25°C for 20 min. K_eq_ was then used to determine unfolding free energy ($\Delta G_{{\text{H}}_{2}\text{O}}^{\text{o}\prime }$) using the following equation: $\Delta G_{{\text{H}}_{2}\text{O}}^{\text{o'}}=-\text{RT}$ ln *K*_eq_ [23], where R is the gas constant in J K^−1^ mol^−1^ and T is the temperature in Kelvin (K). A plot of Δ*G* as a function of the urea molarity follows a linear relationship.

### Protein thermal denaturation analysis using SYPRO orange dye

Protein thermal shift analysis was conducted using SYPRO orange dye (Thermo Fisher Scientific, Waltham, MA, USA) and an Applied Biosystems StepOnePlus real-time PCR instrument (Thermo Fisher Scientific). The assay involved heating the proteins (3 mg/mL) in buffer D with 25× SYPRO orange dye, with a temperature range of 25–99°C and a heating rate of 1°C/min. The thermal denaturation midpoint (*T*_m_ value) was determined using Protein Thermal Shift software v1.4 (Thermo Fisher Scientific).

### Determination of Grx3 activity

The Grx3 activity was measured using a hydroxyethyl disulfide (HED) assay [24]. A mixture of 1 mM GSH (Sigma, St. Louis, MO, USA), 0.4 mM NADPH (Tokyo Chemical Industry, Tokyo, Japan), 2 mM EDTA, 0.1 mg/mL bovine serum albumin, and 0.04 μg yeast glutathione reductase (Sigma) was prepared in buffer D (100 mM Tris·HCl, pH 8.0). An 800 μL aliquot of this mixture was combined with 2.5 mM HEDs (Sigma) and 13.8 μg of SpGrx3. The decrease in absorbance at 340 nm was recorded after 5 min and measured using a Shimadzu UV-1800 spectrophotometer. The enzyme activities of SpGrx3 WT and mutants in the presence of GSH were measured using various GSH concentrations at the optimal temperature. The *K*_m_ and *k*_cat_ values were determined by analyzing the data using a Lineweaver–Burk plot, which provides a double reciprocal representation of the enzymatic reaction kinetics. The activity of the enzyme was expressed as μmol NADPH oxidized per min.

### Circular dichroism (CD) analysis of protein secondary structure

CD spectroscopy in the far-UV range was conducted at 25°C using a JASCO J-1500 spectropolarimeter at the Center for Scientific Instruments, Kyungpook National University (Daegu, South Korea). Protein samples at a final concentration of 0.7 mg/mL in 100 mM Tris·HCl buffer (pH 8.0) were stored at 4°C for 1 h, prior to the CD measurements. The CD spectra were presented as residual ellipticity (mdeg) as a function of wavelength. The secondary structure composition, including the content of α-helix and β-strand, was determined using the Bestsel server [25].

## Results

### Expression and purification of SpGrx3 WT and mutants

**Fig 2:**
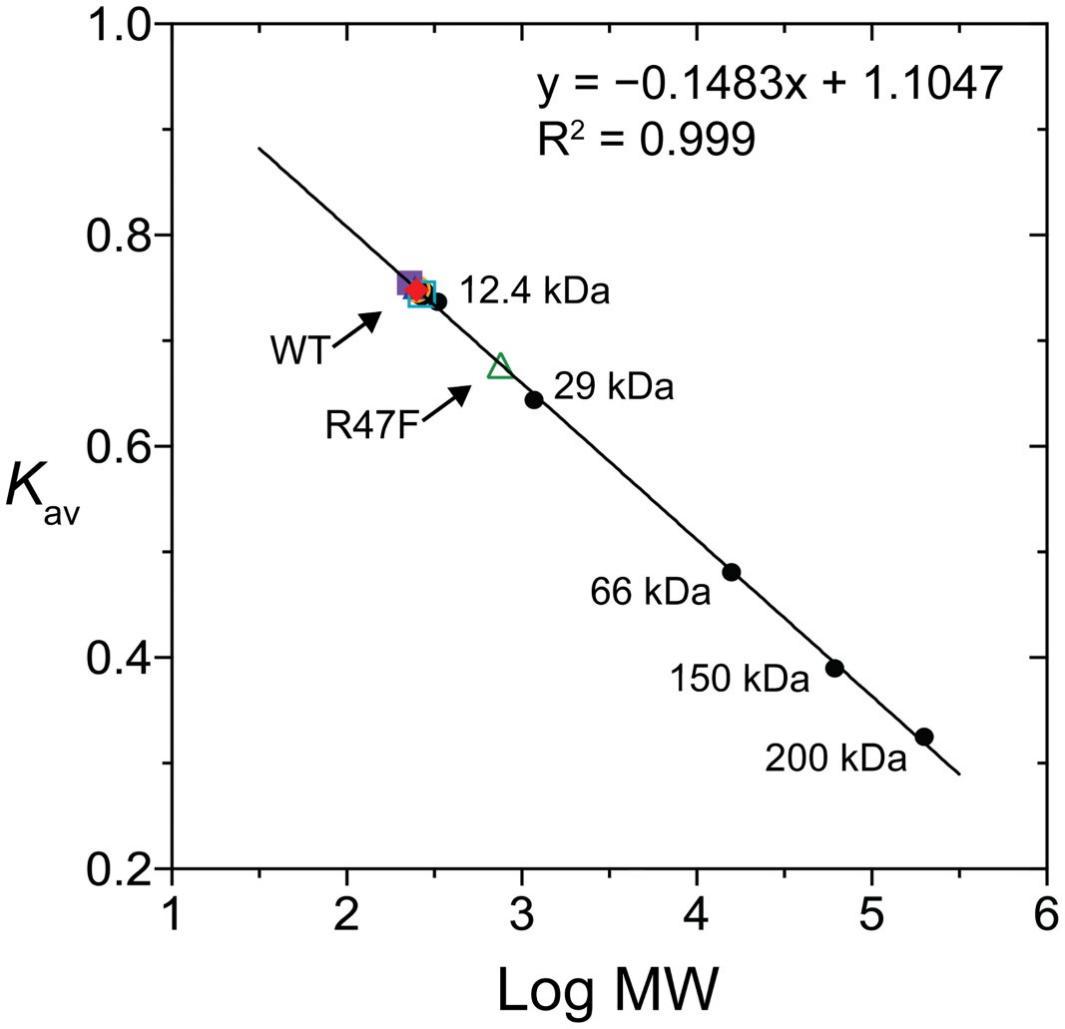
Size-exclusion chromatography analysis. Determination of the MW of native SpGrx3 WT and mutants by size-exclusion chromatography on a Superdex 200 prep grade XK16 column. The arrows indicate that the estimated MW of SpGrx3 WT and other mutants is 11.0 kDa, while the R47F mutant forms a dimer with an MW of 18.0 kDa.

The N-terminal 6×His-tagged recombinant SpGrx3 WT and mutant proteins were successfully expressed as soluble proteins in *E*. *coli* BL21 (DE3). A two-step purification process involving HisTrap affinity chromatography and Capto Q anion-exchange chromatography was employed to achieve homogeneity in the purified protein samples. SpGrx3 WT and mutants showed an MW of 11.0 kDa on SDS-polyacrylamide gel (S1 Fig). In size-exclusion chromatography, the R47F mutant appeared as a dimer of 18.0 kDa, while the WT and other mutants were monomeric (Fig 2 and S2 Fig). These results indicate that Arg47 plays a crucial role in preventing the dimer formation of SpGrx3.

### Effect of mutations on intrinsic fluorescence properties of SpGrx3

**Fig 3:**
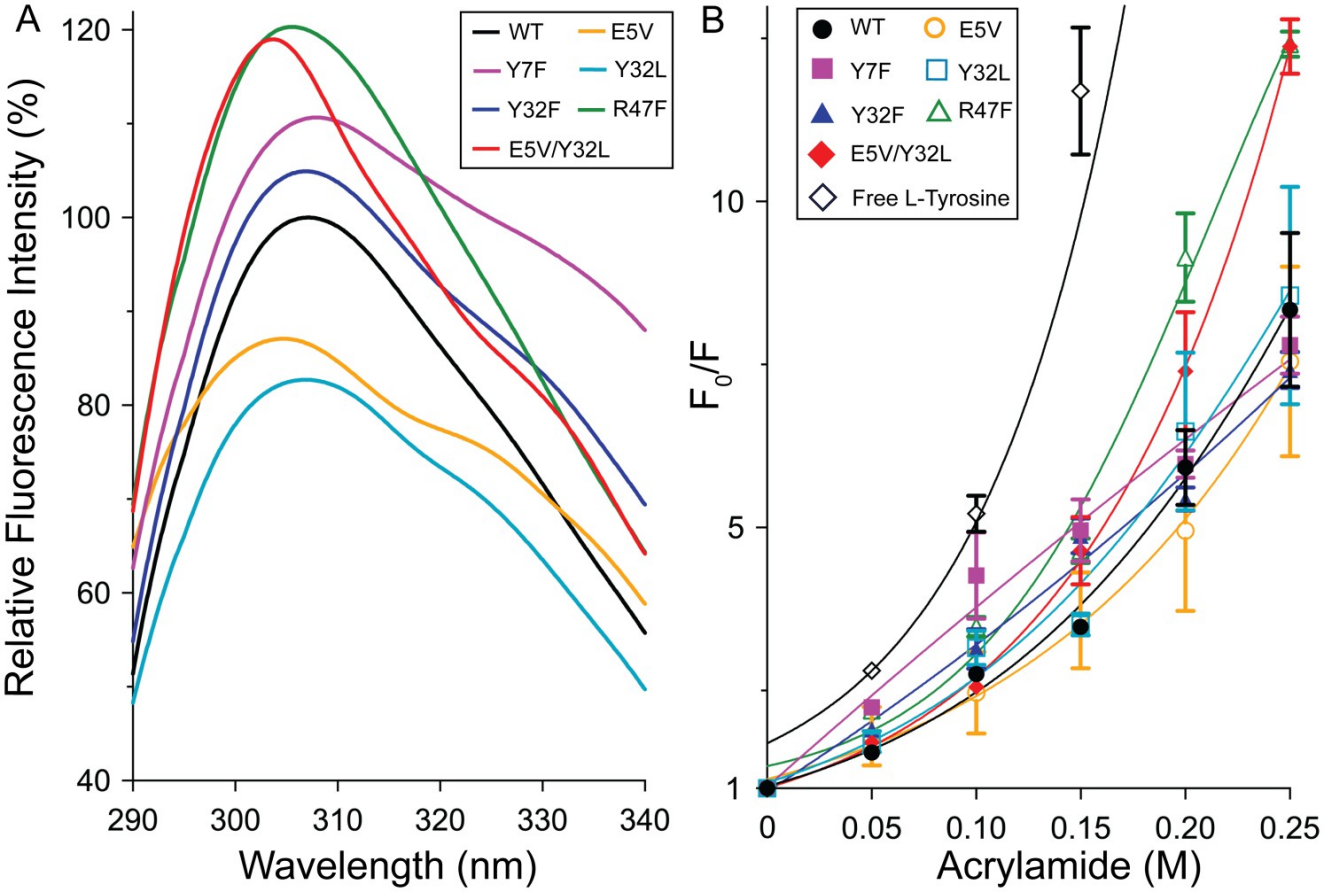
Intrinsic fluorescence and acrylamide Stern–Volmer plot of SpGrx3 WT and mutants. (A) Intrinsic fluorescence at 25°C (excitation at 275 nm). The fluorescence of the WT was normalized to 100%. (B) Acrylamide Stern–Volmer plot. F_0_ represents the maximum fluorescence intensity without acrylamide, while F represents the maximum fluorescence intensity at increasing concentrations (0–0.25 M) of acrylamide. Data presented are the means ± S.D. of three experiments.

Fluorescence spectroscopy was used to measure the intrinsic protein fluorescence of WT and mutant SpGrx3 proteins at 25°C (Fig 3A). Due to the generally lower abundance of aromatic amino acids, especially Trp, in Grx [26], the fluorescence of Tyr was measured. The mutants R47F and E5V/Y32L, which created an increased hydrophobic environment surrounding Tyr7, exhibited the highest fluorescence emission, both showing a 1.20-fold increase compared to WT. Next, the Y7F and Y32F mutants, replacing Tyr with Phe in β1 and β2, respectively, exhibited 1.10- and 1.04-fold increases in fluorescence intensity compared to WT, despite lacking a Tyr residue. This suggests that the aromatic-aromatic interactions involving Phe7-Tyr32 (Y7F) or Tyr7-Phe32 (Y32F) demonstrate enhanced hydrophobicity relative to interactions involving two Tyr residues in WT. On the contrary, the single mutants with disrupted salt bridge and hydrogen bond (E5V) or absent aromatic-aromatic interactions (Y32L) exhibited lower fluorescence emission than WT, indicating the susceptibility of the environment surrounding Tyr7 to water molecules. These results emphasize the importance of hydrophilic amino acids in the aromatic cluster surrounding Tyr7 for reducing hydrophobicity, and the presence of aromatic-aromatic interactions between Tyr7 and Tyr32. The formation of the R47F mutant dimer, as shown in Fig 2, along with its hydrophobic dimer interface, provides support for the observed increase in fluorescence emission.

### Assessment of conformational flexibility in SpGrx3 mutants

The conformational flexibility of SpGrx3 WT and mutant proteins was assessed by analyzing acrylamide-induced quenching of protein fluorescence at 25°C (Fig 3B). The acrylamide Stern–Volmer plot revealed that mutants with disrupted β1-α2 interactions (R47F and E5V/Y32L) exhibited the highest flexibility, despite their hydrophobic β-sheet core observed in Fig 3A. In contrast, mutants with altered β1-β2 interactions (E5V, Y7F, Y32L, and Y32F) displayed similar conformational flexibility to WT. These findings suggest that the β1–α2 interactions involving Glu5, Tyr7, and Arg47 play a significant role in the conformational flexibility of SpGrx3, while the β1–β2 interactions have a lesser impact.

### Thermal shift analysis in SpGrx3 mutants

The *T*_m_ values, representing the temperature at which 50% of the protein is denatured, were determined for SpGrx3 WT and mutants using SYPRO orange-based thermal shift analysis (Table 1 and S3 Fig). The WT exhibited a *T*_m_ of 52.04°C. Hydrophilic-to-hydrophobic substitutions (E5V, Y7F, Y32F, and R47F) in β1, β2, and α2 increased *T*_m_ values by 5.5–7.6°C compared to WT. Particularly, replacing Tyr with Phe (Y7F and Y32F mutants) further enhanced *T*_m_ values for aromatic interactions compared to Tyr7-Tyr32 interactions in WT. In contrast, Y32L and E5V/Y32L mutants, lacking aromatic interactions with Tyr7, exhibited *T*_m_ values 9.1 and 8.5°C lower than WT, respectively. Interestingly, despite disrupted β1-α2 interactions and the resulting increased conformational flexibility, the R47F mutant showed a 5.5°C higher *T*_m_ value (57.49°C) than WT, suggesting that the hydrophobic dimer interface of the R47F mutant contributes to its enhanced thermal stability. These findings emphasize the importance of decreased hydrophobicity in the aromatic cluster for maintaining the structure of SpGrx3. Moreover, our data demonstrate the role of aromatic interactions between β1 and β2 in contributing to the protein’s thermal stability.

**Table 1:** *T*_m_ values of SpGrx3 WT and mutants.

	*T*_m_ (°C)
WT	52.04 ± 0.23
E5V	58.37 ± 0.22
Y7F	58.95 ± 0.39
Y32L	42.87 ± 0.23
Y32F	59.67 ± 0.09
R47F	57.49 ± 0.04
E5V/Y32L	43.45 ± 0.17

The data presented are the means ± S.D. of three experiments.

### Tertiary structural changes in SpGrx3 mutants probed with bis-ANS

**Fig 4:**
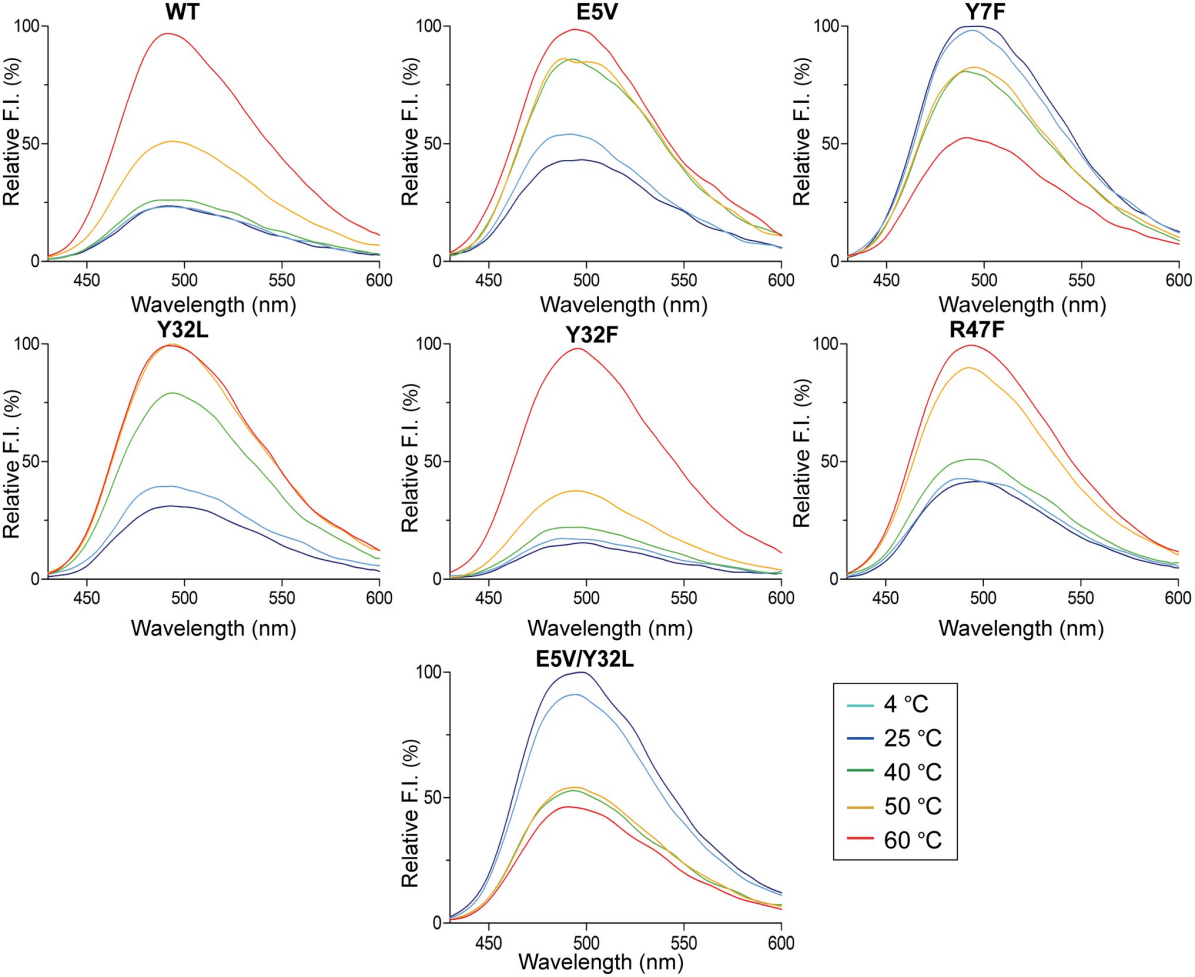
Bis-ANS fluorescence of SpGrx3 WT and mutants. The temperature-induced unfolding of SpGrx3 and its mutants was assessed by measuring bis-ANS fluorescence after incubating the proteins at various temperatures (4–60°C) for 1 h (excitation at 385 nm). The highest fluorescence of each protein was set as 100%. Data presented are the means of three experiments.

Fluorescence spectroscopy was also employed to assess the tertiary structure stability of SpGrx3 WT and mutants (Fig 4). Bis-ANS, a hydrophobic probe, was used to monitor changes in the exposure of hydrophobic regions in SpGrx3, which lacks Trp residues and has low intrinsic fluorescence, after a 1-h incubation from 4 to 60°C. The WT maintained its thermal stability in the 4–40°C range and it was denatured from 50°C. Among the mutants, Y7F and E5V/Y32L, which exhibited increased hydrophobicity within the β1-strand, formed aggregates and showed reduced fluorescence emission starting from 40°C. Conversely, E5V and R47F, with disrupted β1-α2 interactions, and Y32L, with disrupted β1-β2 interactions, exhibited unstable structures and underwent denaturation at 40°C. In contrast, Y32F displayed increased thermal stability compared to WT, maintaining its native form up to 60°C. These findings suggest that excessive hydrophobicity within the β1-strand can lead to protein aggregation in SpGrx3, while substituting Tyr32 with Phe in β2 contributes to a more stable structure.

### Conformational stability evaluation in SpGrx3 mutants

**Fig 5:**
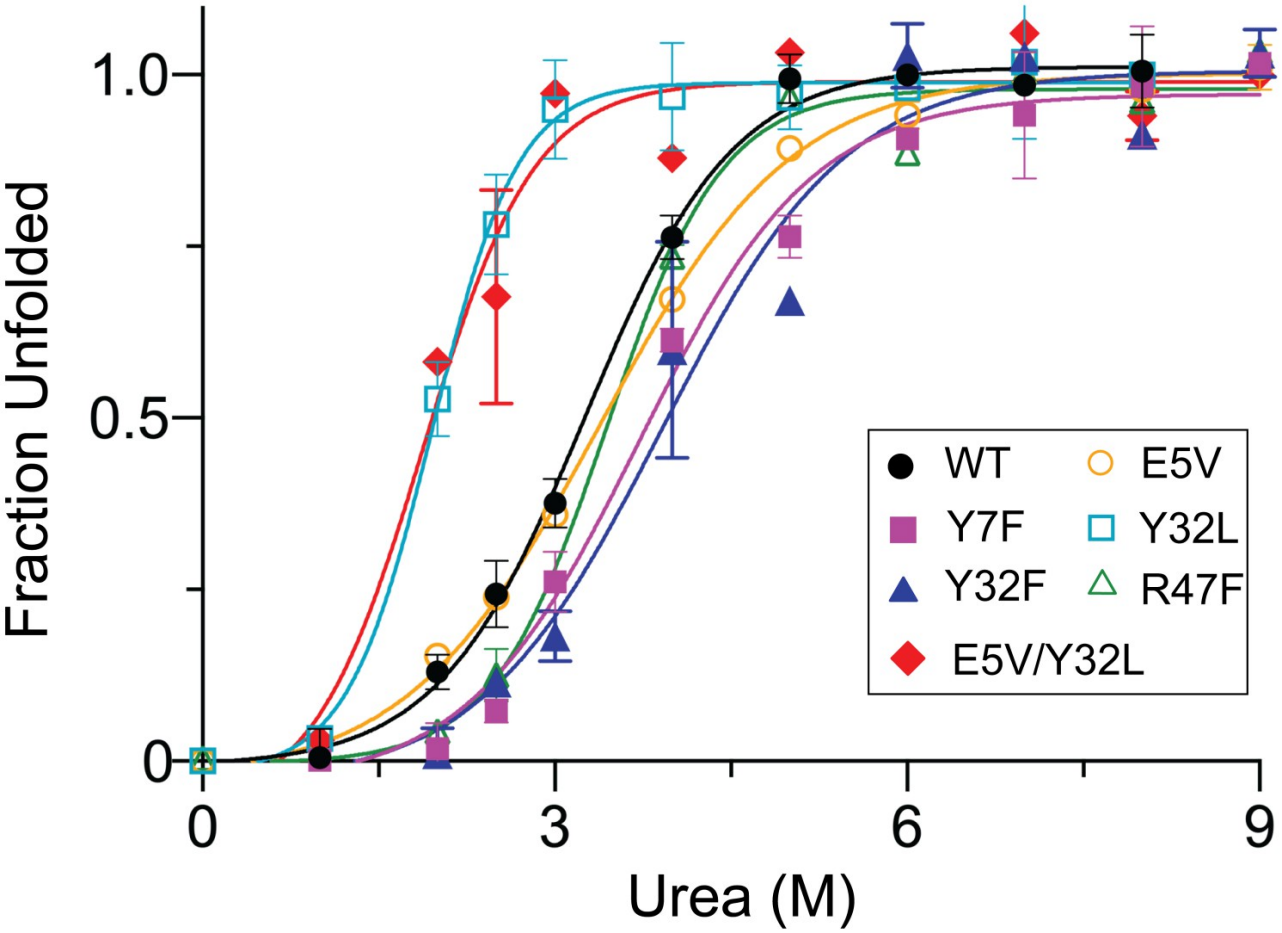
The urea-induced unfolding of SpGrx3 WT and mutants. Fluorescence spectra were measured after incubating the proteins with different concentrations of urea (0–9 M) at 25°C for 20 min (excitation at 275 nm). Data presented are the means ± S.D. of three experiments.

The conformational stability of SpGrx3 WT and mutants was assessed by urea-induced protein unfolding using fluorescence spectroscopy at 25°C (Fig 5). Mutants with increased hydrophobicity (E5V, Y7F, Y32F, and R47F) showed stability in urea compared to WT. The increased stability of the Y7F and Y32F mutants is attributed to the enhanced β1-β2 aromatic interactions through the substitution of Tyr with the more hydrophobic Phe. In contrast, the Y32L and E5V/Y32L mutants, which lack these aromatic interactions, displayed reduced stability, particularly at lower urea concentrations. Interestingly, the β1-α2 interactions, whether hydrogen bond or salt bridge, had no significant impact on urea stability, as demonstrated by the increased stability of E5V and R47F mutants. These findings underscore the importance of hydrophobic and aromatic-aromatic interactions in the β-sheet core for the conformational stability of SpGrx3.

The unfolding-free energy ($\Delta G_{{\text{H}}_{2}\text{O}}^{\text{o}\prime }$) was calculated to assess protein stability in the absence of urea (Table 2). The mutants exhibited a reduction in $\Delta G_{{\text{H}}_{2}\text{O}}^{\text{o}\prime }$ in the following order: Y32F > Y7F > R47F > E5V > WT > Y32L and E5V/Y32L. The aromatic-aromatic interactions in WT contributed approximately –1.5 kcal/mol to the free energy change in water, indicating their stabilizing effect, compared to Y32L and E5V/Y32L mutants lacking these interactions. The contribution of aromatic-aromatic interactions to stability is generally observed within the range of –0.6 to –1.3 kcal/mol [27]. On the other hand, the unfolding-free energy analysis showed that hydrophobic interactions in E5V, Y7F, Y32F, and R47F mutants ranged from –0.1 to –1.6 kcal/mol. These results highlight the key role of hydrophobic interactions in contributing to the stability of SpGrx3 in urea.

**Table 2:** Stability parameters for SpGrx3 WT and mutants.

Protein	[D]_1/2_ ^a^	*m* ^b^	$\Delta G_{H_{2}O}^{o'}$ ^c^
	(M)	(kcal mol^-1^ M^-1^)	(kcal mol^-1^)
WT	3.33 ± 0.24	0.69 ± 0.04	2.30 ± 0.01
E5V	3.43 ± 0.05	0.69 ± 0.03	2.37 ± 0.07
Y7F	4.31 ± 0.11	0.77 ± 0.02	3.31 ± 0.01
Y32L	1.46 ± 0.07	0.54 ± 0.01	0.79 ± 0.06
Y32F	4.37 ± 0.02	0.89 ± 0.01	3.89 ± 0.02
R47F	3.75 ± 0.19	0.70 ± 0.03	2.61 ± 0.01
E5V/Y32L	1.60 ± 0.10	0.48 ± 0.04	0.77 ± 0.05

^a^ Concentration of urea at which the unfolding transition midpoint is observed.

^b^ Proportionality constant between free energy and urea concentration.

^c^ Free energy of unfolding extrapolated to zero denaturants. Data presented are the means ± S.D. of three measurements.

### Secondary structure analysis of SpGrx3 mutants

**Fig 6:**
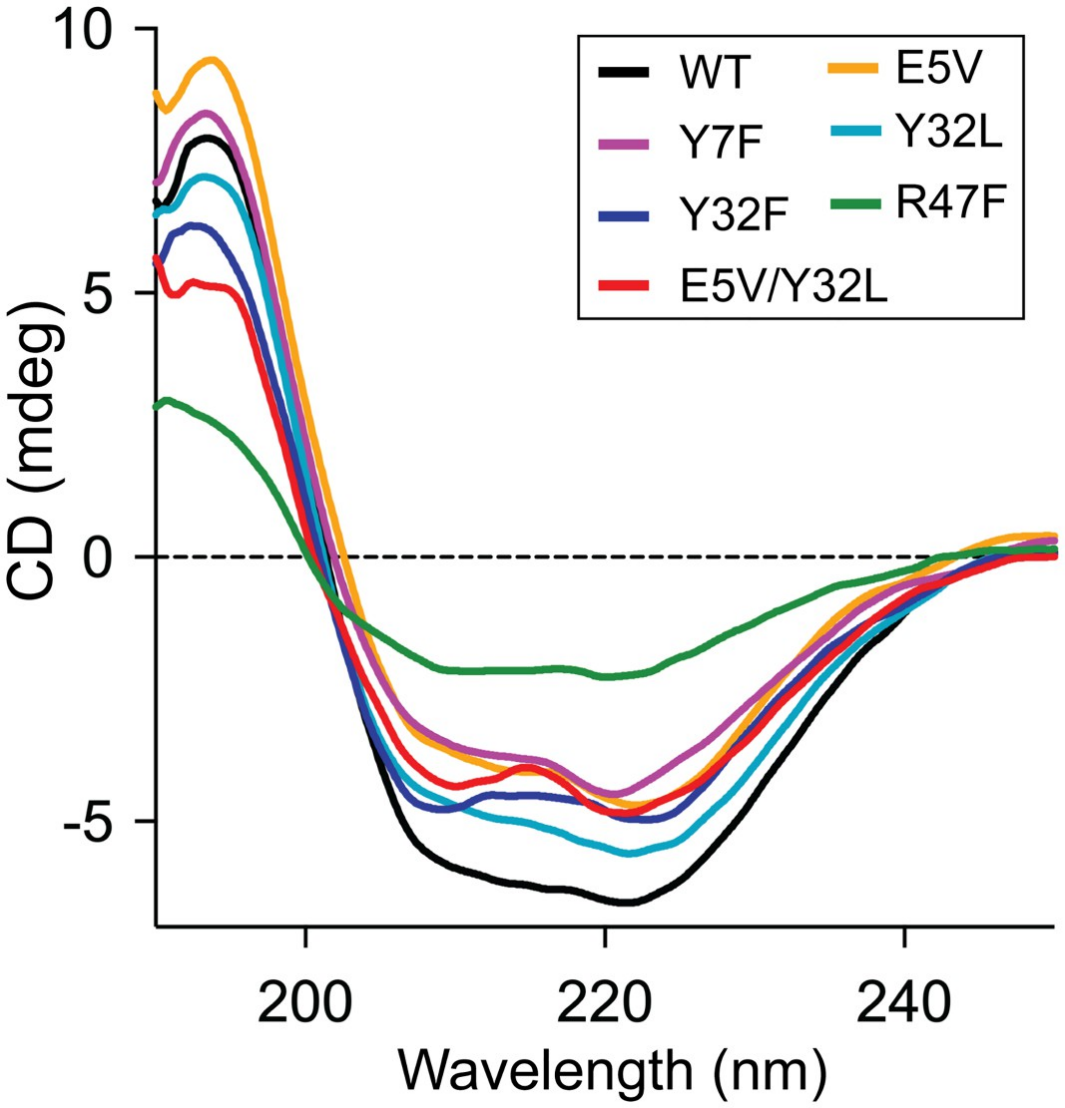
Far UV-CD spectra of SpGrx3 WT and mutants. The CD spectra were measured at 25°C following a 1-h incubation of the proteins at 4°C.

The effects of mutations on the secondary structure of SpGrx3 were evaluated using far-UV CD spectroscopy at 25°C (Fig 6). The WT exhibited 27.1% α-helix and 18.4% β-strand content (S3 Table). All mutants displayed a reduced α-helical content compared to WT. Among the mutants, R47F showed the lowest α-helical content of 7.7% and the highest β-strand content of 43.8%. These findings suggest that the substitution of hydrophilic residues with hydrophobic ones (Y7F, Y32L, Y32F, and E5V/Y32L) led to a decrease in α-helical content and an increase in β-strand content. In contrast, E5V and R47F, which disrupted the Glu5-Arg47 salt bridge connecting β1 and α2, exhibited a further reduction in α-helical content. Our findings suggest that the hydrogen bond network and the salt bridge comprising the aromatic cluster play crucial roles in the secondary structure of SpGrx3.

### Enzyme kinetics investigation in SpGrx3 mutants

The kinetic profiles of SpGrx3 WT and mutants were determined using GSH as a substrate and NADPH as a cofactor at their respective optimal temperatures (Table 3 and S4 Fig). WT showed a *K*_m_ value of 4.01 mM and a *k*_cat_ value of 314.14 min^−1^. The hydrophilic-to-hydrophobic substitutions in general resulted in a more rigid active-site structure, leading to reduced *K*_m_ values, except for the Y32F and E5V/Y32L mutants. Interestingly, the Y32F mutant, with a hydrophilic residue (Tyr7) in the β1-strand, exhibited similar *K*_m_ values to WT. In contrast, the E5V/Y32L mutant, which displayed the most flexible structure among the mutants, showed 1.74-fold higher *K*_m_ values than WT. Furthermore, the substitutions led to a decrease in the catalytic rate compared to WT. Specifically, the E5V and R47F mutants, where the β1-α2 interaction was disrupted, exhibited the lowest catalytic rate. These findings suggest that hydrophilic amino acids in the aromatic cluster are important for maintaining a flexible conformation of the active site for substrate binding. In contrast, the β1-α2 interactions, particularly the salt bridge, play a crucial role in preserving conformational flexibility and modulating the catalytic rate of the enzyme.

**Table 3:** Kinetic parameters of SpGrx3 WT and mutants.

	*K*_m_ (mM)	*k*_cat_ (min^-1^)	*k*_cat_/*K*_m_ (min^-1^mM^-1^)
WT	4.01 ± 0.08	314.14 ± 5.16	78.31 ± 0.26
E5V	2.24 ± 0.48	92.14 ± 25.41	40.66 ± 2.33
Y7F	2.03 ± 0.12	160.41 ± 7.54	79.12 ± 1.29
Y32L	2.40 ± 0.07	159.82 ± 3.73	66.16 ± 0.36
Y32F	4.22 ± 0.21	220.93 ± 10.67	52.28 ± 0.07
R47F	2.39 ± 0.04	84.18 ± 1.58	35.22 ± 0.13
E5V/Y32L	6.98 ± 0.87	362.12 ± 20.01	51.88 ± 0.73

The data presented are the means ± S.D. of three experiments.

## Discussion

The canonical Trx-fold is highly conserved across evolution, despite the functional diversity observed within the Trx-fold superfamily [6, 7, 28]. Trx and Grx are small redox proteins that play important roles in maintaining cellular redox homeostasis [29]. However, there are structural differences between Trx, which functions as a substrate of Trx reductase, and Grx, which acts as an enzyme [30]. Grxs, in particular, exhibit a more flexible active site and an opposite electrostatic surface potential, which is a major determinant of substrate specificity in the Trx-fold family [26, 31, 32]. While both Trxs and Grxs have a low frequency of aromatic amino acids, Grxs are characterized by the absence of Trp residues [26]. Furthermore, Trx and Grx show distinct arrangements of aromatic residues in their aromatic clusters. Trx and Grx4 have a tetrahedral arrangement [12, 33], while Grx3, as observed in this study, exhibits a parallel arrangement. In SpGrx3, the substitution of Arg47 with Phe leads to the formation of a tetrahedral aromatic cluster (Y7, Y32, F47, and F58). This unique aromatic cluster configuration in SpGrx3 underscores the evolutionary divergence between Grx3 and Trx, representing two distinct protein families [34, 35].

The central β-sheet of Trx maintains its rigidity throughout evolution [36–38]. In *E*. *coli* Trx (EcTrx), the hydrophobic interactions between β2 and β4 are crucial for maintaining the active-site conformation, participating in the initial folding step of Trx, and connecting the two large folding units (βαβαβ and ββα) [39–41]. Sequence comparison of Trx-fold proteins reveals the conservation of two aromatic residues on the central β-sheet, specifically β2 and β4 in Trx and β1 and β3 in Grx3 (Fig 1D). In our study, hydrophobic substitutions within the β1-strand increased intrinsic protein fluorescence and induced aggregation formation, highlighting the importance of reduced hydrophobicity in the β1-strand in maintaining the Trx-fold in Grx3. Specifically, in the Y7F and Y32F mutants, where Phe replaced Tyr in β1 or β2, we observed stronger aromatic-aromatic interactions compared to Grx3 WT with the Tyr-Tyr pair, indicating a higher degree of hydrophobic clustering between the aromatic side chains [42]. Furthermore, we observed unfavorable aggregation formation in the Y7F and E5V/Y32L mutants at elevated temperatures, underscoring the detrimental effects of excessive hydrophobic interactions within the β1-strand of SpGrx3 (Fig 4). These findings overall emphasize the significant role of β1-β2 aromatic interactions in enhancing the stability of the β-sheet core in Grx3. Interestingly, in GrxA (*Synechocystis* sp. PCC 6803), a T11Y mutation at the end of the β1-strand resulted in the formation of insoluble protein [43].

Despite originating from a psychrophilic bacterium, SpTrx displays remarkable thermal stability with a *T*_m_ value of 73.4°C [12]. In contrast, SpGrx3, an oxidoreductase with a modified secondary structure characterized by one less α-helix and one less β-strand [44, 45], resulting in reduced β-sheet hydrophobicity compared to Trx, exhibits lower thermal stability (*T*_m_ value of 52.04°C) and enhanced conformational flexibility. This is evident from the 6.3-fold higher F_0_/F value observed in the acrylamide Stern–Volmer plot compared to SpTrx (S5 Fig). The presence of additional loop regions in Grxs also contributes to their overall structural flexibility [26]. Contrary to the central β-sheet rigidity observed in SpTrx, SpGrx3 exhibits flexibility in the short α2-helix and a reduction in hydrophobicity within the aromatic cluster. Moreover, the loop preceding the α2-helix in psychrophilic Grx3 members has fewer hydrogen bonds compared to orthologous EcGrx3 (S6 Fig), further supporting the role of the α2-helix in the flexible structure of Grx3. These findings align with previous studies on EcTrx, where the short α3-helix exhibited increased flexibility during the evolutionary transition from ancestral Trxs [36, 37, 46]. Comparison of EcTrx with resurrected LBCA (last bacterial common ancestor) and Precambrian Trx (LPBCA, representing the origins of photosynthetic bacteria) revealed that the loss of charge-dipole interaction involving Lys88 in LBCA or Arg88 in LPBCA in β5 contributed to the increased flexibility of the short α3-helix in EcTrx [36, 47]. These observations highlight the evolutionary processes involving the short α-helix in shaping the structural and functional characteristics of Grx3.

The presence of Tyr32 in β2 is unique to SpGrx3, while other Grx3 members typically have aliphatic residues (Val, Leu, or Ile) at this position (Fig 1D). The β1-β2 aromatic interactions play a crucial role in the stability of the central β-sheet in SpGrx3. However, as an oxidoreductase, SpGrx3 is expected to possess a flexible structure in other regions. Structural analysis of thermophilic and mesophilic Grx3 members revealed the existence of a salt bridge between α1 and β2, located on the opposite side of the central β-sheet (S6 Fig). In contrast, SpGrx3 lacks this salt bridge due to the substitution of Arg/Lys with Leu (S6 Fig). These results suggest that α1-β2 interactions in orthologs adapted to warmer temperatures serve a similar role to the β1-β2 aromatic interactions in stabilizing the central β-sheet in SpGrx3. This mechanism is analogous to the central β-sheet stability in Trx, wherein the α3-helix exhibits increased flexibility, while the α4-helix displays enhanced rigidity [36]. This alteration in conformational dynamics in distal regions is a commonly observed mechanism in protein structures [37] and directly influences the functional evolution of enzymes [48].

The far-UV CD spectra revealed notable changes in secondary structure for mutants with increased hydrophobicity, characterized by a decrease in α-helical content and an increase in β-strand content (Fig 6 and S3 Table). Particularly, the R47F mutant exhibited significant alterations, with 7.7% α-helix and 43.8% β-strand content. These structural changes in the R47F dimer likely contributed to the reorganization of the secondary structure and the observed increase in β-strand content [49, 50]. Additionally, our structural analysis showed that *E*. *coli* Grx1, which shares the canonical Trx-fold and has 37% sequence identity with *E*. *coli* Grx3, features a Lys residue in the short α2-helix [44, 51], highlighting the crucial role of positively charged residues in maintaining the secondary structure (S7 Fig).

Grx3 members also feature a His residue in β4, which contributes to the stabilization of the cis-Pro loop through hydrogen bonding with a Gln residue [33]. Protonated His exhibits stronger hydrogen bonding capabilities compared to neutral groups [52, 53]. His in β4 dominates as a unique feature among Grx3 members, independent of habitat temperature. On the other hand, the aromatic cluster in class II redox-inactive Grx4 members adopts a tetrahedral geometry of aromatic residues resembling Trxs [33]. In Grx4, the aromatic residue in β3 (the third amino acid following cis-proline) plays a significant role in modulating the stability and flexibility of the cis-Pro loop, depending on the habitat temperature [33]. Additionally, class II Grxs exhibit a distinctive feature with an elongation of five amino acids in the loop preceding the N-terminal active site Cys, setting them apart from class I Grxs [8, 54].

In conclusion, Grx3 undergoes structural modifications in its β-sheet core, resulting in a unique aromatic cluster configuration with reduced hydrophobicity and the presence of Arg/Lys in α2. The disruptions in β1-β2 and β1-α2 interactions have distinct effects on the stability and flexibility of Grx3. This study provides valuable insights into the relationship between variations in the Trx-fold structure and functional versatility.

## Supporting information

S1 Table List of primers for cloning into a TA vector and site-directed mutagenesis. (PDF)

S2 Table Inverse Stern–Volmer quenching constant, *K*D-1 (M). (PDF)

S3 Table Content of α-helix and β-strand in SpGrx3 WT and mutants. (PDF)

S1 Fig SDS-polyacrylamide gel electrophoresis of SpGrx3 WT and mutants. (PDF)

S2 Fig Size-exclusion chromatography of SpGrx3 WT and mutants. (PDF)

S3 Fig Melting temperatures of SpGrx3 WT and mutants. (PDF)

S4 Fig Apparent optimum temperatures of SpGrx3 WT and mutants. (PDF)

S5 Fig Acrylamide Stern–Volmer plot of WT SpGrx3 and SpTrx. (PDF)

S6 Fig Structural comparison of α1–α2 (A) and α1–β2 (B) side-chain interactions in class I Grx3 members across thermophiles to psychrophiles. (PDF)

S7 Fig Sequence comparison of Grx1 and Grx3 members. (PDF)
