# Supplementary material for: Unraveling the roles of aromatic cluster side-chain interactions on the structural stability and functional significance of psychrophilic Sphingomonas sp. glutaredoxin 3
Source: PLoS One. 2023 Aug 31;18(8):e0290686. doi: 10.1371/journal.pone.0290686 (PMC10470887; doi:10.1371/journal.pone.0290686)
Supplement: S1 Table — (PDF) [file pone.0290686.s001.pdf]

S1 Table. List of primers for cloning into a TA vector and site-directed mutagenesis.

		Primer sequences
WT	Forward	5´-cggcatatgtccaagatcgaaatct-3´ (*Nde* I site underlined)
	Reverse	5´-gaattctcacacgccgagcag-3´ (*EcoR* I site underlined)
E5V	Forward	5´-gtccaagatcGTAatctacacc-3´
	Reverse	5´-ggtgtagatTACgatcttggac-3´
Y7F	Forward	5´-atcgaaatcTTCaccaaag-3´
	Reverse	5´-ggctttggtGAAgatttc-3´
Y32L	Forward	5´-cggaagagTTAgacatcac-3´
	Reverse	5´-gtgatgtcTAActcttccg-3´
Y32F	Forward	5´-gtcacgccggaagagTTTgacatc-3´
	Reverse	5´-gcccatcgtgatgtcAAActcttcc- 3´
R47F	Forward	5´-cgaaatgctcgagTTCgcc- 3´
	Reverse	5´-cggccattggcGAActc- 3´

Mutated nucleotides are shown in capital letters. The E5V/Y32L double mutant was constructed using the primer for E5V based on the Y32L template.
