# Supplementary material for: Unraveling the roles of aromatic cluster side-chain interactions on the structural stability and functional significance of psychrophilic Sphingomonas sp. glutaredoxin 3
Source: PLoS One. 2023 Aug 31;18(8):e0290686. doi: 10.1371/journal.pone.0290686 (PMC10470887; doi:10.1371/journal.pone.0290686)
Supplement: S2 Table — (PDF) [file pone.0290686.s002.pdf]

S2 Table. Inverse Stern–Volmer quenching constant, K_D^{-1} (M).

	K_D^{-1} (M).
SpGrx3 WT	0.037 ± 0.002
E5V	0.041 ± 0.009
Y7F	0.037 ± 0.003
Y32L	0.049 ± 0.010
Y32F	0.041 ± 0.002
R47F	0.022 ± 0.000
E5V/Y32L	0.023 ± 0.001
SpTrx WT	0.232 ± 0.010

K_D^{-1} is the acrylamide concentration at which 50% of the fluorescence intensity is quenched.
