# Supplementary material for: Unraveling the roles of aromatic cluster side-chain interactions on the structural stability and functional significance of psychrophilic Sphingomonas sp. glutaredoxin 3
Source: PLoS One. 2023 Aug 31;18(8):e0290686. doi: 10.1371/journal.pone.0290686 (PMC10470887; doi:10.1371/journal.pone.0290686)
Supplement: S3 Table — (PDF) [file pone.0290686.s003.pdf]

S3 Table. Content of α-helix and β-strand in SpGrx3 WT and mutants.

	α-helix (%)	β-strand (%)
WT	27.1	18.4
E5V	16.7	29.5
Y7F	10.7	28.1
Y32L	16.3	30.2
Y32F	17.3	25.9
R47F	7.7	43.8
E5V/Y32L	13.9	31.1
