# Supplementary figures and images for: Unraveling the roles of aromatic cluster side-chain interactions on the structural stability and functional significance of psychrophilic Sphingomonas sp. glutaredoxin 3
Source: PLoS One. 2023 Aug 31;18(8):e0290686. doi: 10.1371/journal.pone.0290686 (PMC10470887; doi:10.1371/journal.pone.0290686)

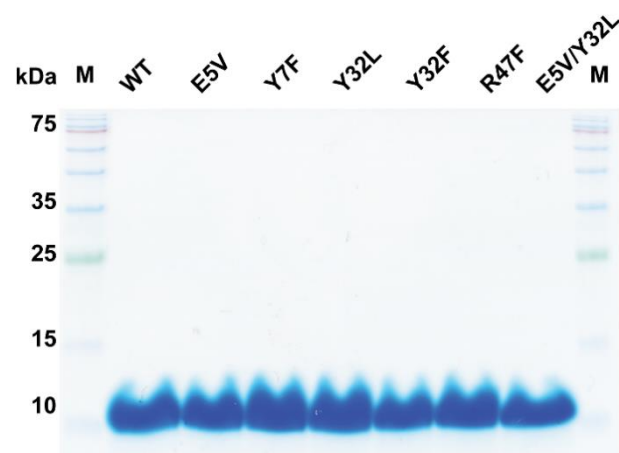

**S1 Fig. SDS-polyacrylamide gel electrophoresis of SpGrx3 WT and mutants. M, marker.**

Supplement: S1 Fig — (PDF) [file pone.0290686.s004.pdf]
