# Supplementary material for: Unraveling the roles of aromatic cluster side-chain interactions on the structural stability and functional significance of psychrophilic Sphingomonas sp. glutaredoxin 3
Source: PLoS One. 2023 Aug 31;18(8):e0290686. doi: 10.1371/journal.pone.0290686 (PMC10470887; doi:10.1371/journal.pone.0290686)
Supplement: S2 Fig — (PDF) [file pone.0290686.s005.pdf]

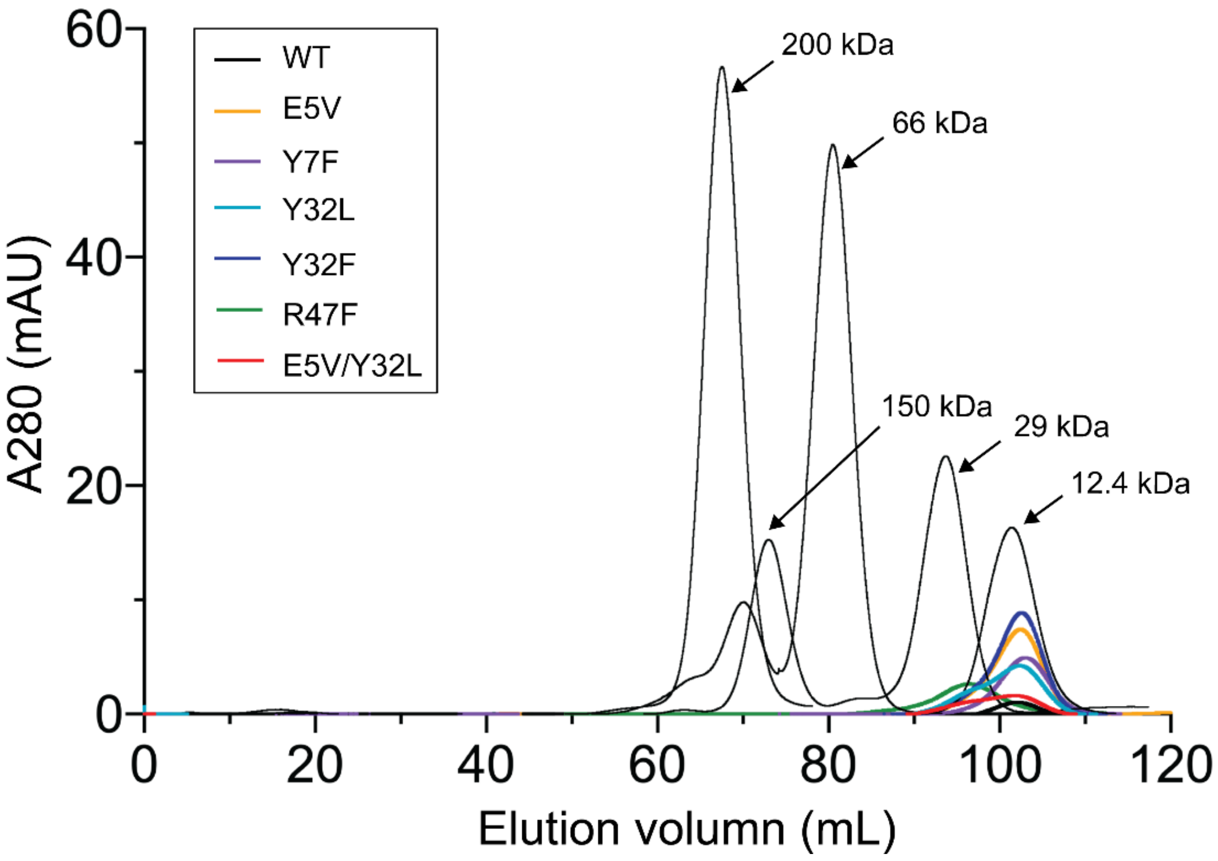

S2 Fig. Size-exclusion chromatography of SpGrx3 WT and mutants. Protein molecular weights were determined using a Superdex 200 prep grade XK16 column in buffer D (50 mM Tris·HCl and 50 mM NaCl, pH 8.0). The black line represents protein molecular weight standards, including β-amylase (sweet potato, 200 kDa), alcohol dehydrogenase (yeast, 150 kDa), albumin (bovine serum, 66 kDa), carbonic anhydrase (bovine erythrocytes, 29 kDa), and cytochrome C (horse heart, 12.4 kDa).
