# Supplementary material for: Unraveling the roles of aromatic cluster side-chain interactions on the structural stability and functional significance of psychrophilic Sphingomonas sp. glutaredoxin 3
Source: PLoS One. 2023 Aug 31;18(8):e0290686. doi: 10.1371/journal.pone.0290686 (PMC10470887; doi:10.1371/journal.pone.0290686)
Supplement: S3 Fig — (PDF) [file pone.0290686.s006.pdf]

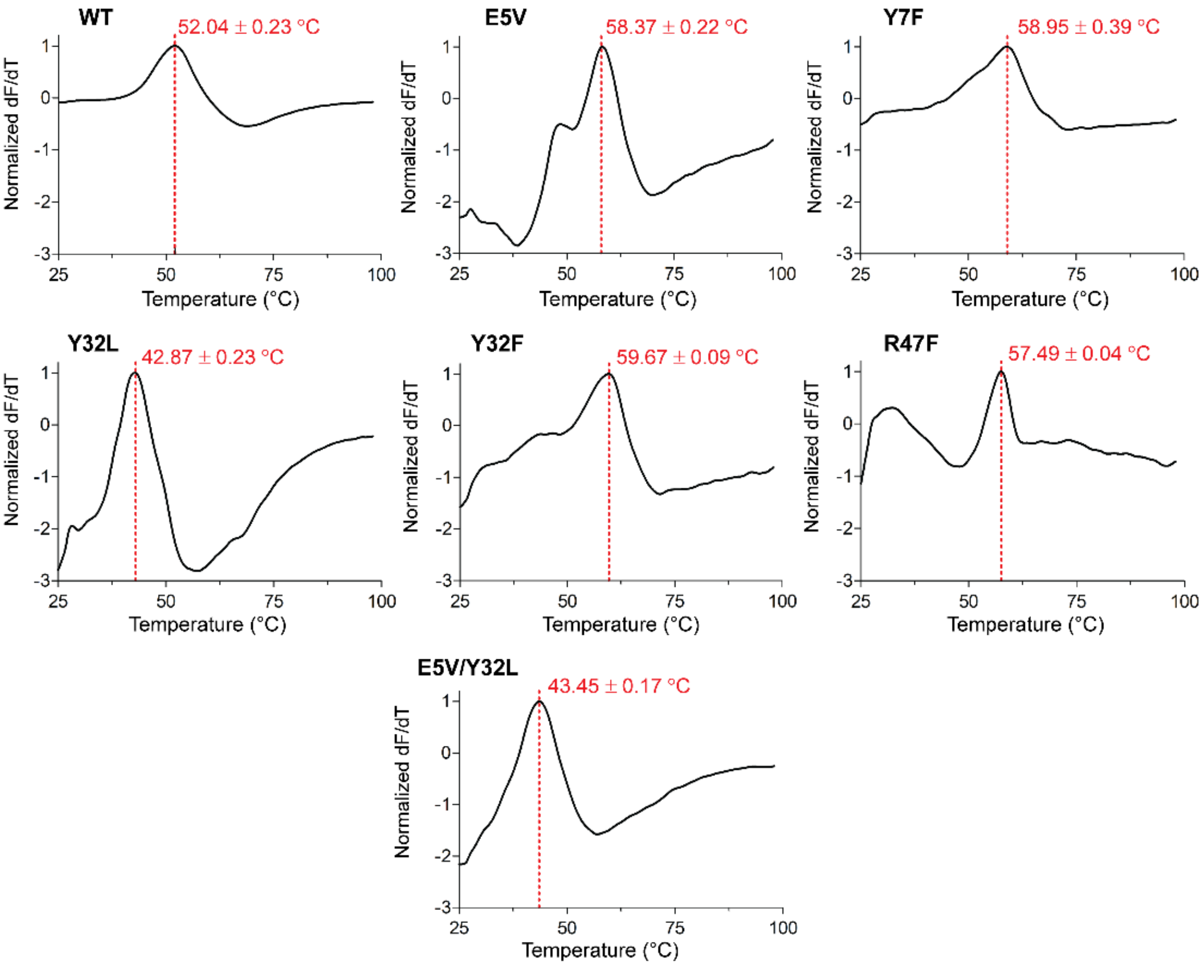

S3 Fig. Melting temperatures of SpGrx3 WT and mutants. The melting temperatures were measured using SYPRO orange dye-based thermal shift analysis in the 25–99 °C range in 1 °C/min increments. Red dotted vertical lines indicate the derivative T_m values. The derivative dF/dt represents the rate of change of the fluorescence signal (F) with respect to time (t) during thermal denaturation. The temperature at which the maximum rate of the peak derivative occurs corresponds to the midpoint of the thermal unfolding transition (T_m). In our analysis, we set the highest fluorescence signal as 1 for normalization. The data are presented as the means ± S.D. of three experiments.
