# Supplementary material for: Unraveling the roles of aromatic cluster side-chain interactions on the structural stability and functional significance of psychrophilic Sphingomonas sp. glutaredoxin 3
Source: PLoS One. 2023 Aug 31;18(8):e0290686. doi: 10.1371/journal.pone.0290686 (PMC10470887; doi:10.1371/journal.pone.0290686)
Supplement: S4 Fig — (PDF) [file pone.0290686.s007.pdf]

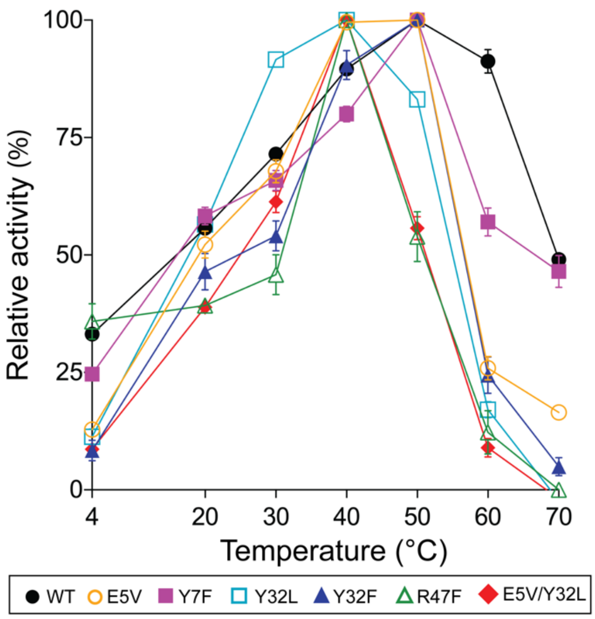

S4 Fig. Apparent optimum temperatures of SpGrx3 WT and mutants. The activity at the optimum temperature of each protein was set as 100%. The data are presented as the means ± S.D. of three experiments.
