# Supplementary material for: Unraveling the roles of aromatic cluster side-chain interactions on the structural stability and functional significance of psychrophilic Sphingomonas sp. glutaredoxin 3
Source: PLoS One. 2023 Aug 31;18(8):e0290686. doi: 10.1371/journal.pone.0290686 (PMC10470887; doi:10.1371/journal.pone.0290686)
Supplement: S6 Fig — (PDF) [file pone.0290686.s009.pdf]

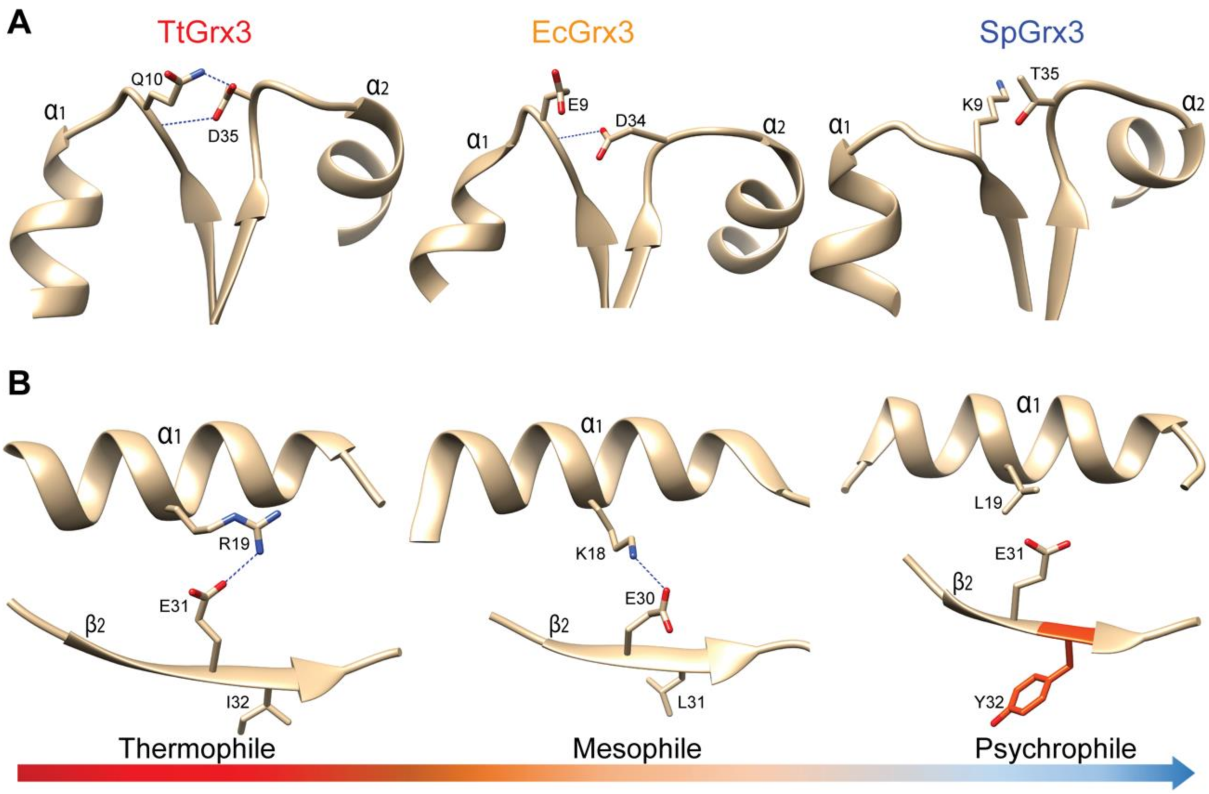

S6 Fig. Structural comparison of α1–α2 (A) and α1–β2 (B) side-chain interactions in class I Grx3 members in TtGrx3 (thermophile), EcGrx3 (mesophile), and SpGrx3 (psychrophile). The crystal structure of EcGrx3 (PDB ID: 1FOV) and the Alpha-Fold 3D model structures of TtGrx3 and SpGrx3 were visualized and analyzed by ChimeraX software (versions 1.6).
