# Supplementary material for: Unraveling the roles of aromatic cluster side-chain interactions on the structural stability and functional significance of psychrophilic Sphingomonas sp. glutaredoxin 3
Source: PLoS One. 2023 Aug 31;18(8):e0290686. doi: 10.1371/journal.pone.0290686 (PMC10470887; doi:10.1371/journal.pone.0290686)
Supplement: S7 Fig — (PDF) [file pone.0290686.s010.pdf]

```
                             α2
EcGrx1  VDIRAEGITKEDLQQKAGKPVETV 59
KqGrx1  VDIHAEGISKADLEKTVGKPVETV 59
PaGrx1  VDIHAEGITKEDLSKSVGKPVETV 59
EhGrx1  VDIHAEGITKADLEKTVGKPVETV 59
EcGrx3  LPIDGNAAKREEMIKRSG--RTTV 53
CjGrx3  IAVDRDRMRWDEMVARSG--RKTV 53
PeGrx3  IRVDLEPALRAEMVRRTG--RRTV 54
PmGrx3  IDVQSAP-VRSEMVSRSG--RRTV 52
        : :        ::    *    **
```

S7 Fig. Sequence comparison of Grx1 and Grx3 members. The mark in yellow color indicates the highly conserved positively charged residues on the α2-helix in both Grx1 and Grx3. The sequences are EcGrx1 (*Escherichia coli*, 1GRX), KqGrx1 (*Klebsiella quasipneumoniae*, EMR24818.1), PaGrx1 (*Pasteurellaceae* sp. NI1060, AOF52359.1), EhGrx1 (*Enterobacter hormaechei*, SSI77961.1), EcGrx3 (*E. coli*, 1FOV), CjGrx3 (*Caldichromatium japonicum*, QIK38813.1), PeGrx3 (*Pelomicrobium methylotrophicum*, TXF13737.1), and PmGrx3 *(Pseudomonas mandelii*, WP_140678632.1).
